# A model of HIV drug resistance driven by heterogeneities in host immunity and adherence patterns

**DOI:** 10.1186/1752-0509-7-11

**Published:** 2013-02-04

**Authors:** Anna Bershteyn, Philip A Eckhoff

**Affiliations:** 1 Epidemiological Modeling Group, Intellectual Ventures Laboratory, Washington, USA

## Abstract

**Background:**

Population transmission models of antiretroviral therapy (ART) and pre-exposure prophylaxis (PrEP) use simplistic assumptions – typically constant, homogeneous rates – to represent the short-term risk and long-term effects of drug resistance. In contrast, within-host models of drug resistance allow for more detailed dynamics of host immunity, latent reservoirs of virus, and drug PK/PD. Bridging these two levels of modeling detail requires an understanding of the “levers” – model parameters or combinations thereof – that change only one independent observable at a time. Using the example of accidental tenofovir-based pre-exposure prophyaxis (PrEP) use during HIV infection, we will explore methods of implementing host heterogeneities and their long-term effects on drug resistance.

**Results:**

We combined and extended existing models of virus dynamics by incorporating pharmacokinetics, pharmacodynamics, and adherence behavior. We identified two “levers” associated with the host immune pressure against the virus, which can be used to independently modify the setpoint viral load and the shape of the acute phase viral load peak. We propose parameter relationships that can explain differences in acute and setpoint viral load among hosts, and demonstrate their influence on the rates of emergence and reversion of drug resistance. The importance of these dynamics is illustrated by modeling long-lived latent reservoirs of virus, through which past intervals of drug resistance can lead to failure of suppressive drug regimens. Finally, we analyze assumptions about temporal patterns of drug adherence and their impact on resistance dynamics, finding that with the same overall level of adherence, the dwell times in drug-adherent versus not-adherent states can alter the levels of drug-resistant virus incorporated into latent reservoirs.

**Conclusions:**

We have shown how a diverse range of observable viral load trajectories can be produced from a basic model of virus dynamics using immunity-related “levers”. Immune pressure, in turn, influences the dynamics of drug resistance, with increased immune activity delaying drug resistance and driving more rapid return to dominance of drug-susceptible virus after drug cessation. Both immune pressure and patterns of drug adherence influence the long-term risk of drug resistance. In the case of accidental PrEP use during infection, rapid transitions between adherence states and/or weak immunity fortifies the “memory” of previous PrEP exposure, increasing the risk of future drug resistance. This model framework provides a means for analyzing individual-level risks of drug resistance and implementing heterogeneities among hosts, thereby achieving a crucial prerequisite for improving population-level models of drug resistance.

## Background

Quantitative analysis of the risk of HIV drug resistance is important on both an individual and a population level, especially when the “real-world” use of drugs may differ significantly from the more ideal setting of randomized controlled trials. Examples of non-ideal “real-world” situations include poor adherence, late entry into therapy, drug stockouts, unauthorized re-distribution of antiretrovirals, and interruption of multi-drug regimens in which one drug has a longer half-life, which creates an interval of “effective monotherapy” when only this drug is present at high levels [1].

Interest in models of drug resistance has increased recently due to the addition of pre-exposure prophylaxis (PrEP) to the world’s HIV prevention toolkit [2]. Clinical trials have demonstrated that PrEP with tenofovir disoproxil fumarate (TDF), combined TDF and emtricitabine (FTC), and tenofovir vaginal gel can reduce the risk of HIV acquisition [3-6]. No PrEP regimen prevented transmission entirely, though increased adherence correlated with increased protection against HIV. This creates a risk of accidental PrEP use during “breakthough” infections, until the individual is diagnosed with HIV and PrEP is discontinued. Additionally, there is risk of accidental initiation of PrEP by infected individuals due to faulty testing or early “window period” testing. In addition to their use in PrEP, TDF and FTC are also found in two popular single-pill, once-daily fixed-dose combination therapies (Atripla and Complera) as well as the fixed-dose “quad” pill recently approved by the FDA. Thus, there is concern as to whether resistance caused by PrEP could threaten the ability to use two of the safest available drugs available in a convenient, single-dose, once-daily regimen.

Mathematical models of ART and PrEP have been used to assess the risk of drug resistance on the individual and population level [7]. However, state-of-the-art population models have failed to capture heterogeneities in the risk of drug resistance among individuals, due to the disparity in model detail between population-level and within-host models.

Population-level models of PrEP and resistance tend to focus on specific conditions of HIV transmission, with a majority of recent oral PrEP models focusing on heterosexual generalized epidemics [8-10], and others on concentrated epidemics among men who have sex with men [11]. Of the small number of population PrEP models that include drug resistance, only one has considered reversion of resistance by assuming that resistance and reversion occur at fixed rates for the treated and post-treated subpopulations, respectively [10,11]. Recent models of combination ART that account for resistance and reversion treat these similarly [12,13]; though one model used stochastic numerical methods as a proxy for variability in acquisition and transmission of resistance, these are still assumed to occur at a fixed rate [13] with no consideration for heterogeneous host biology and behavior.

Within-host models have provided some mechanistic insights into HIV progression, drug efficacy, and the risk of resistance. Nowak and Bangham formulated a model of virus dynamics that includes mutation [14], and has inspired dozens of variations, such as mechanisms of viral escape arising from competition among quasispecies-specific cytotoxic T lymphocyte (CTL) [15] and antibody [16] responses. A common application of these models is predicting the relationship between dosage and acquisition of resistance: a quantity not well-characterized in clinical studies [17]. Very recently, a detailed within-host model similar to the one presented here was used to propose a mechanism for drug resistance during HAART that includes a protease inhibitor, proposing a relationship between adherence level and risk of treatment failure [18]. This and other models [19] have assumed that adherence is primarily characterized by the percentage, not the pattern, of doses taken. Other models have incorporated time-correlated field data [20] and to explore the impact of “clustering” dropped doses on the drug resistance outcome [21]. Measuring the true patterns of drug adherence can be challenging [22-25], but models can represent a range of possible behaviors *in silico* to understand the effects of different possible patterns.

The host immune response is another source of heterogeneity that, as we will show, contributes substantially to the dynamics of resistance and reversion. Though a wealth of biological studies have revealed the complex relationship between immunity and virologic control, few models have explored the effect of viral replication and immunity on drug efficacy [26,27].

Ultimately, population models must capture the important outcomes of within-host heterogeneities and react appropriately to a range of assumptions about viral fitness, host immune response, drug pharmacology, and adherence. Here, we use the example of tenofovir-based PrEP to explore the insights that can be gained from considering such heterogeneities in a combined model of within-host virus dynamics, drug pharmacokinetics/pharmacodynamics (PK/PD), and patterns of drug adherence.

## Methods

We constructed a model that combines the pharmacokinetic/pharmacodynamic outcome of time-varying drug adherence with the dynamics of competition between drug-resistant mutants. Hypotheses or data about drug adherence patterns are provided as input for the simulation, and the model predicts the time-varying populations of CD4+ T cells and virus (WT and drug-resistant) in the form of plasma RNA and integrated DNA. The model is deterministic, but can be driven stochastically through the choice of adherence pattern.

**Figure 1:**
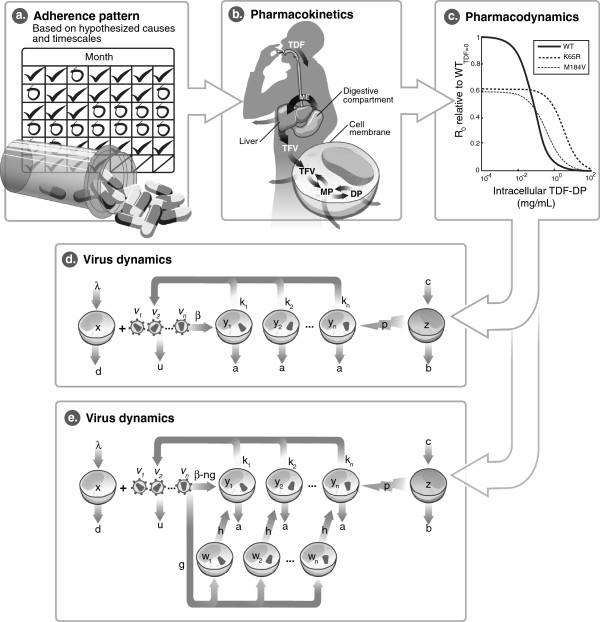
**Model schematic. (a)** The model first assumes an adherence pattern, which is translated into a series of taken or missed doses over time. Doses, if taken, are assumed to be taken at a specified interval, e.g., daily for TDF. **(b)** A pharmacokinetic model of TDF translates the series of doses into a time-varying concentration of TDF-DP in the active intracellular compartment. By including the longer half-life of intracellular TDF-DP compared to TFV, this model captures the “pharmacologically forgiving” properties of TDF. **(c)** The relationship between the TDF-DP concentration and the replication of WT or mutant virus is assumed to be a Hill function following the median-effect model. WT is better able to replicate in the absence of drug, whereas the drug-resistant mutants are able to replicate at higher drug concentrations. *K65R* has higher fitness than *M184V* at all drug concentrations, and thus is expected to predominate over *M184V* in all simulations. **(d)** The basic virus dynamics model, governed by Equations 2–5, is depicted graphically. The additional factors of forward- and back-mutation due to error in reverse transcription, not depicted here, are shown in Equations 6–7. **(e)** The expanded virus dynamics model, which includes a latently infected cell compartment *w*, is depicted here and in Equations 11–12.

Figure 1 illustrates the components of the model and the way in which they are linked. First, an adherence pattern is created based on a hypothesis about the timescales of adherent and non-adherent time intervals. For example, adherence could be assumed to be random in time, periodic with a given duration of adherence and non-adherence, or stochastic based on a Markov model described in detail below.

Next, a detailed pharmacokinetics model is used to calculate the time-varying concentration of the active form of the drug in the relevant compartment, given the sequence of doses that were taken over time. For oral TDF, the model includes bioavailability, partitioning across the cell membrane, and the two phosphorylation steps required to create the active diphosphate form of the drug.

A pharmacodynamics model is used to translate the drug concentration into an effect on the replicative capacity of the WT and mutant viral quasispecies.

Finally, a dynamic model of viral replication responds to the changes in replicative capacity caused by fluctuating drug concentrations. Here, uninfected CD4+ target cells (denoted *x*) are infected by a given viral quasispecies (*v*_*i*_ for quasispecies *i,* e.g., *v*_*WT*_ and *v*_*mutant*_) to form a productively infected cell (*y*_*i*_). Infected cells can be eliminated by virus-specific CTLs (*z*). In an alternative version of this model (Figure 1b), infection can yield long-lived latently infected cells (*w*_*i*_) with probability *g*, or productively infected cells (*y*_*i*_) with probability *1-g*. Latently infected cells produce no virus and are not susceptible to CTLs until they become activated into productively infected cells at a slow rate, *h*.

### Model parameters

All parameter values and associated references are listed in Table 1. Except for studies of acute infection in the absence of drug, the model was initialized at presumed steady-state values of *x*_*ss*_, *y*_*ss*_, *v*_*ss*_, and *z*_*ss*_ for numerical integration. Simulations of acute infection dynamics were initialized at *x* = 10^6^, *v*_*i*_ = 1, and *y*_*i*_ = *w*_*i*_ = *z* = 0.

**Table 1:** Parameter values

							
**Parameter**	**Value**	**Units**	**Ref**	**Parameter**	**Value**	**Units**	**Ref**
***v***_***SS***_	51,000	*copies mL*^*-1*^	[32]	*m*_***WT***_	1.139	*Unitless*	[46]
**λ**	10^5^	*cells mL*^*-1*^ *day*^*-1*^	[32]	*m*_***K65R***_	1.126	*Unitless*	[46]
***b***	0.027	*day*^*-1*^	[32]	***IC***_**50*,WT***_	0.050*	*μg mL*^*-1*^	[46]
***d***	0.1	*day*^*-1*^	[32]	***IC***_**50*,K65R***_	2.2*	*μg mL*^*-1*^	[46]
***u***	5	*day*^*-1*^	[32]	**K**_**a**_	14.64	*day*^*-1*^	[41]
***a***	0.5	*day*^*-1*^	[32]	**K**_**e**_	9.6	*day*^*-1*^	[41]
***c***_**1**_	1000	*cells mL*^*-1*^ *day*^*-1*^	[32]	**K**_**1f**_	9.6	*day*^*-1*^	[41]
**β **	2*f* × 10^5^	*mL count*^*-1*^ *day*^*-1*^	[32]	**K**_**1b**_	30.3	*day*^*-1*^	[41]
***f***	Variable (Equation 9)	*Unitless*		**K**_**2f**_	270.7	*day*^*-1*^	[41]
***h***	0.002	*day*^*-1*^	[41]	**K**_**2b**_	95.5	*day*^*-1*^	[41]
***w***_***ss***_***/y***_***ss***_**(for*g*)**	0.39	*Unitless*	[72]	**K**_**m**_	24000	*day*^*-1*^	[41]
***k***_***WT***_	100/*f*	*day*^*-1*^	[32]	**K**_**c**_	1.1	*day*^*-1*^	[41]
***k***_***K65R***_	61.054/*f*	*day*^*-1*^	[46]	**f**_**plasma-bound**_	0.07	*Unitless*	[41]
**H**	1800	*Unitless*	[42]	* Concentration in plasma.			

### Adherence patterns

A hypothesis or data set related to adherence is converted to a bit string representing whether the dose is taken or missed at each time in the dosing schedule. The sequence could potentially be informed by field data, e.g., if a time-correlated data set such as electronic monitoring of bottle opening were used [24,25].

We begin with a simple periodic model in which the frequency of switching between adherent and non-adherent states is altered while maintaining the same total fraction of missed doses. However, when considering potential causes of poor adherence, neither periodic nor random/unstructured missing of doses is fully realistic: there are various reasons for poor adherence with different intrinsic time scales, which can introduce temporal correlations in adherence patterns. As an example of an intermediate between the fully periodic and fully random models, we also implemented a Markov model of discrete drug-taking and drug-missing states, which we call “adherence states.” Each state has a characteristic dwell time, analogous to models of packet loss in communication channels [28,29]. The time spent in the *i*^th^ adherence state before switching to a different state was calculated using the Gillespie stochastic simulation algorithm [30,31] as

(1) $$\mathrm{\Delta t}=\frac{-1}{\sum_{j\in J_{i}\;}r_{i\to j}}\mathrm{In}\left(\mathrm{rand}\right)$$

where *J*_*i*_ represents the set of possible states that could be entered from *i*, *r*_*i* → *j*_ is the rate of transitioning from state *i* to state *j*, and *rand* is a random number uniformly distributed between 0 and 1. After time ∆*t*, the next state was chosen among possible states *J*_*i*_ proportionally to the relative magnitude of the transition rates *r*_*i* → *j*_. Finally, the states were discretized into a binary sequence of taken or missed doses by determining whether the individual was in an ART-taking or ART-missing state at the time of each dose.

### Virus dynamics and immune response

We used a classic model of virus dynamics [14,32,33] extended to include immune pressure by CTLs [32-34] as shown in Figure 1b. For uninfected CD4+ target cells *x*, infected cells *y*, free virus *v*, and CTLs *z*, the competing dynamics of multiple quasispecies, indexed by *i*, are governed by:

(2) $$\dot{x}=\lambda -\mathrm{dx}-\underset{i}{\sum }\mathrm{\beta x}v_{i}$$

(3) $$\dot{y}{\;}_{i}=\mathrm{\beta x}v_{i}-ay_{i}-py_{i}z$$

(4) $$\dot{v}{\;}_{i}=k_{i}y_{i}-uv_{i}-\mathrm{\beta x}v_{i}$$

(5) $$\dot{z}=c-\mathrm{bz}$$

We assumed that the population of free viruses and cell-incorporated viruses interact with a common pool of uninfected target cells, and that immune pressure *p* was exerted equally on all infected cells. (This assumption would break down if the drug resistance mutation occurred in an epitope targeted by two CTLs that may be present at significantly different levels or subject to different levels of immune regulation.) The steady-state frequency of HIV-specific CTLs *z*_ss_ = c/b was set to 37 cells/uL, consistent with phenotypic analysis of HIV-infected human peripheral blood [35,36]. We assumed z grows at rate *c* independently of *z, y*, or the product *zy*, the dynamics of which are described elsewhere [32-34]. We explored this system under varying *p* because this is a known heterogeneity among hosts: viremia is elevated [37] or suppressed [38] in the presence of certain HLA-restricted CTL subtypes.

Single-point mutants form during infection of a target cell *x* by wild type (*WT*) drug-susceptible virus *v*_*WT*_, due to reverse transcription with a per-position mutation probability *q*. We assumed that each mutant could revert to wild-type with the same probability. The dynamics of the mutant (Equation 6) and wild-type (Equation 7) infected cell populations are affected by this assumption as follows:

(6) $$\dot{y}{\;}_{i,\;i\neq \mathrm{WT}}=\left(1-q\right)\mathrm{\beta x}v_{i}-ay_{i}-py_{i}z+\mathrm{q\beta x}v_{\mathrm{WT}}$$

(7) $$\begin{array}{l} \dot{y}{\;}_{\mathrm{WT}}=\left(1-\underset{i\neq \mathrm{WT}}{\sum }q\right)\mathrm{\beta x}v_{\mathrm{WT}}-ay_{\mathrm{WT}}-py_{\mathrm{WT}}z\\ \;+\underset{i\neq \mathrm{WT}}{\sum }\mathrm{q\beta x}v_{i} \end{array}$$

whereas the growth of target cells, free virions, and CTLs were still governed by Equations 2, 4, and 5, respectively.

At steady-state

$$\left(\dot{x}={\dot{y}}_{i}={\dot{v}}_{i}=\dot{z}=0\right)$$

with the approximation that − *βxv*_*i*_ ≈ 0 when calculating

$$\dot{\nu }$$

(negligible depletion of virus due to infection of new target cells) and the assumption of negligible mutation, Equations 2–5 can be solved to estimate the steady-state viral load of the dominant quasispecies [32]:

(8) $$v_{\mathrm{ss}}=\frac{\mathrm{\lambda k}}{\left(a+\frac{c}{b}p\right)}-\frac{d}{\beta }$$

Increasing the magnitude of the CTL response *p* reduces *v*_*ss*_. To modulate *p* while preserving *v*_*ss*_ and the clearance rates of each species, a multiplier *f* can be applied to modify the original infection rate *β*_0_ into an adjusted rate *β  = β*_0_*f*, and modify the original virus production rate *k*_0_ into an adjusted rate *k = k*_*0*_*/f*. The value of *f* is calculated from the original parameter values and the desired value of *v*_*ss*_ using the equation

(9) f=1vssλk0a+cbp−dβ 0

Changing *f* alone preserves the overall dynamics of the system, including the basic reproductive ratio of the virus [32]:

(10) $$R_{0}=\frac{\mathrm{\beta \lambda k}}{\left(a+\frac{c}{b}p\right)\mathrm{du}}$$

We investigated the effect of changing *p* with and without a compensating change in *f* that preserves *v*_*ss*_ at the mean value measured in discordant heterosexual couples in Zambia, 51,000 copies/mL [39].

To incorporate latency into the model for a subset of the simulations, we added an optional latent compartment *w* with latency rate *g* and reactivation rate *h* (for clarity, shown here using the simplified equations without mutation):

(11) $$\dot{y}{\;}_{i}=\left(\beta -g\right)xv_{i}+hw_{i}-ay_{i}-py_{i}z$$

(12) $$\dot{w}{\;}_{i}=\mathrm{gx}v_{i}-hw_{i}$$

For the dominant subtype, the steady-state level of latently infected cells is approximately:

(13) wss=ghxssvss=ghβ λ−adukβ

The total body load of activated CD4^+^ T cells containing incorporated HIV DNA has been measured in lymph nodes and blood and estimated to be approximately 3.1 x 10^7^, compared to approximately 1.2 x 10^7^ resting CD4^+^ T cells with incorporated viral DNA [40]. We fixed the ratio *w*_*ss*_*/y*_*ss*_, with

(14) $$y_{\mathrm{ss}}=\frac{\lambda }{\left(a+\frac{c}{b}p\right)}-\frac{\mathrm{du}}{\mathrm{\beta k}}=\frac{u}{k}v_{\mathrm{ss}}$$

This relationship allowed us to calculate *g* for a given value of *h* to ensure *w*_*ss*_*/y*_*s*_ = 0.39 using

(15) $$g=h\frac{\mathrm{\beta k}}{au}\frac{w_{\mathrm{ss}}}{y_{\mathrm{ss}}}$$

In the latency simulations shown, we set the latently infected cell reactivation rate *h* to 0.002 days^-1^, representing the long-lived component of the latently infected cell reservoir [41]. The simplifying assumptions made by this implementation of latency include (1) a constant low rate of gradual reactivation, in lieu of the wide range of re-activation rates possible from different cell subtypes, and (2) the production of a single activated cell by each latently infected cell, neglecting proliferation in the process of re-activation.

### Pharmacokinetics

We implemented a previously reported model by Dixit and Perelson [42] that captures the more “pharmacologically forgiving” nature of intracellular TDF-DP as compared to shorter-lived TDF in plasma. Tenofovir partitions across cell membranes and, inside cells, is phosphorlyated twice into its active antiviral form, TDF-diphosphate (TDF-DP) [43]. Importantly, the half-life of intracellular TDF-DP is 10-fold higher than that of TDF in plasma [44,45]. We used model equations and parameters published by Dixit and Perelson [42]. Briefly, we modeled the drug concentration in the digestive compartment (*C*_*d*_), extracellular compartment (*C*_*e*_), intracellular compartment (*C*_*c*_), intracellular singly phosphorylated state (*C*_*cp*_), and the active, intracellular double phosphorylated state (*C*_*cpp*_) using the following equations:

(16) $$\dot{C_{d}}=-K_{a}C_{d}$$

(17) $$\dot{C_{e}}=K_{a}C_{d}-K_{e}C_{e}$$

(18) $$\begin{array}{l} \dot{C_{c}}=K_{m}\max(0,\left(1-m)HC_{e}-C_{c}\right)-K_{c}C_{c}\\ \;-K_{1f}C_{c}+K_{1b}C_{\mathrm{cp}} \end{array}$$

(19) $$\begin{array}{l} \dot{C_{\mathrm{cp}}}=-K_{c}C_{\mathrm{cp}}+K_{1f}C_{c}-K_{1b}C_{\mathrm{cp}}-K_{2f}C_{\mathrm{cp}}\\ \;+K_{2b}C_{\mathrm{cpp}} \end{array}$$

(20) $$\dot{C_{\mathrm{cpp}}}=-K_{c}C_{\mathrm{cpp}}+K_{2f}C_{\mathrm{cp}}-K_{2b}C_{\mathrm{cpp}}$$

The rate constants for this model, also taken from Dixit and Perelson [41], are listed in Table 1.

### Pharmacodynamics

The pharmacodynamics component of the model takes as input the time-varying drug concentration in its active form (*C*_*cpp*_) and determines its time-varying effect on viral replication. We modeled only the single mutants known to confer drug resistance using recently reported dose–response data from single-cycle replication studies [46]. Mutation is assumed to incur a fitness penalty that reduces the virus production rate *k*. In the case of the *K65R* mutant, this rate is reduced by 39% at zero drug concentration. Multiple-position mutants were not modeled.

TDF-DP concentrations *D(t)* in the intracellular compartment are assumed to reduce *β * through their effect on reverse transcriptase. Each mutant is affected according to the median-effect model [47]

(21) β i=β 11+DtIC50,im

where *IC*_*50*_ is the median-effect dose and *m* is the Hill coefficient. We used values recently reported from an *in vitro* study [46] and multiplied the *IC*_*50*_ by the partition coefficient of TDF across the cell membrane in the pharmacokinetic model [42] to reflect the intracellular activity of TDF-DP.

## Results

### Heterogeneities in infection and immunity

The early transient (acute phase) and steady-state (chronic phase) trajectories of viral load are measurable and vary among individuals, potentially reflecting heterogeneity in underlying viral replication parameter values. To understand the insights that may be gained by including these heterogeneities in our model of adherence/resistance (Figure 1a), we began by identifying the “levers” within the virus dynamics model (Figure 1a and b) that can be used to reproduce these clinically observable heterogeneities. We hypothesized that the immune pressure parameter *p*, alone and in combination with other parameters, could be leveraged to effect these heterogeneities.

Observations of viremia during early HIV infection have revealed an early peak in viral load that is 2 logs (±1 log) higher than the setpoint viral load [48-52]. The time of the acute phase peak has been reported in the range of 12–31 days [53] or 5–19 days [54]. Some models lacking an immune compartment attribute the peak [55] and setpoint [56] viremia exclusively to the depletion of uninfected target cells rather than immune pressure. While such models could still achieve a 2-log reduction in viremia from peak to setpoint, the depletion of uninfected target cells is extreme when this drop is driven by target cell depletion alone, with nearly all CD4+ T cells ending up in the infected state. An immune response component was required to preserve a substantial fraction of uninfected target cells while producing a characteristic acute phase, both of which are features of realistic infection dynamics. Furthermore, correlations between CTLs and viremia in humans [57-59], as well as direct studies of CD8+ T cell depletion in primates [60-62], provide evidence for an important role for CTLs in bringing viral load down to setpoint. Therefore, we chose a model wherein both target cell depletion and CTL-mediated killing contribute to setpoint and acute phase virus dynamics. We then hypothesized that variability in immune response among hosts could, even in a simple model, reproduce the range of possible viral load trajectories among individuals.

**Figure 2:**
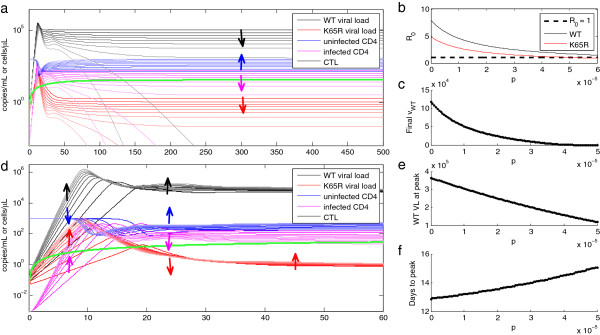
**Influence of CTL efficacy*p*on the dynamics of early infection with changing or constant setpoint viral load. (a)** Copies/mL of WT virus (black) and drug resistant mutant *K65R* (red), and number/μL of CTLs (green), uninfected CD4+ cells/mL (blue) and infected CD4+ cells/mL (magenta), for ten *p* values spaced between 0 and 10^-4^. Lighter shading corresponds to smaller values of *p*, and arrows point in the direction of increasing *p* to show its influence on the trajectories. **(b)** Calculated estimate of the basic reproductive ratio for WT virus and *K65R* mutant as a function of *p*. **(c)** Simulated WT viral load after 500 days, sweeping over *p* while holding all other parameters constant, reveals that the final viral load drops toward zero as *p* reduces the *R*_*0*_ of WT toward 1. **(d)** Increasing *p* and correspondingly decreasing *f* according to Equation 9 maintains the steady-state viral load at 51,000 copies/mL, but raises the acute phase peak and leads to a longer “shoulder” before the viral load reaches setpoint. (Same color scheme as (**a**), but with a timescale of only 60 days to show the details of acute phase dynamics.) The effects on the height and timing of the peak are shown in **(e)** and **(f)**, respectively, over the same range of *p* as in (**b**) and (**c**).

Increasing the immune pressure parameter *p* reduced setpoint viral load with little influence on the acute phase (Figure 2a), which occurs during the ramp-up in CTL frequency and therefore is subject to lower levels of immune pressure compared to steady-state levels. As predicted by Equation 10 and illustrated in Figure 2b, increasing *p* reduces the basic reproductive ratio (*R*_*0*_) of both wild-type and mutant virus. Because mutant virus incurs a higher fitness cost in the absence of drug, the level of *p* required to suppress the mutant virus population (*R*_*0*_ < 1) is lower for the mutant than for the wild-type virus. The steady-state viral load declines with increasing *p*, providing a “lever” to modulate setpoint viral load over the range of physiologically relevant values.

A second, independent “lever” was required to modulate the acute phase viral load trajectory. This is because viral load during the acute phase is not predictive of setpoint viral load [53,63] nor rate of AIDS progression [50,63], although AIDS progression is correlated with setpoint viral load [64-66] and symptoms such as fever, vomiting, diarrhea, and headache during acute infection [50].

Frequent longitudinal sampling of patients with an estimated date of infection has revealed three equally common patterns of viral load trajectories: rapid decline to setpoint, a slow “shoulder” with gradual decline to setpoint, and an initial interval of viral suppression followed by a rebound of viremia, which takes over 90 days to return to setpoint [53]. With this range of biological variability in mind, we searched for a “lever” that can vary the shape (height, timing, and “shoulder”) of the acute phase viral load trajectory without changing setpoint viral load.

Sensitivity analysis of the virus dynamics model (Additional file 1: Table S1) found the acute phase peak time to be most sensitive to β and k, and the acute phase peak height to be most sensitive to k (closely followed by λ and u). Dividing *k* and multiplying β by a factor *f* preserves the number of virions produced with each replication cycle, but modulates the setpoint viral load. To keep the setpoint viral load constant, we used *f* to offset the change in setpoint viral load caused by *p*, changing the two parameters together as per Equation 9. We found that these counterbalanced parameters modulate the peak time, magnitude, and settling time of the peak of acute phase viremia while maintaining a constant setpoint viral load (Figure 2c-d). A biological interpretation of *f* might be ascribed to variability in non-CTL components of the host immune response, such as the relative magnitude of the neutralizing antibody response, which reduces β, and innate immune responses such as antiviral factors, which reduce *k*.

Both “levers” described here rely on the immune pressure parameter *p*, an expected source of heterogeneity among hosts. We next asked how *p* influences the response to antiretroviral therapy and the risk of resistance, using the context of misused tenofovir-based PrEP as a relevant scenario.

### Resistance and reversion of mutations: effect of immune response

Human studies of TDF monotherapy have not agreed on a characteristic timescale for the emergence of resistance. Two human studies of daily TDF, lasting 21 days [67] and 28 days [68] respectively, did not find evidence of resistance. In the rhesus macaque SHIV model, TDF monotherapy elicited *K65R* mutations with associated viral rebound in all animals within 2 to 12 weeks (median 4 weeks) of therapy initiation [69]. This implies that the human trials were likely too short to detect the emergence of resistance. We therefore used our model to simulate longer timescales of drug exposure.

**Figure 3:**
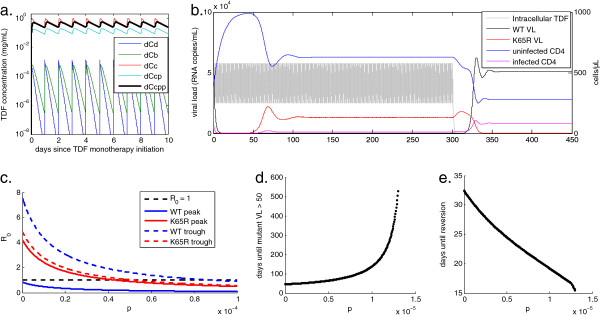
**Effect of immune pressure on rate of development and reversion of drug resistance. (a)** Concentration of digestive (*C*_*d*_), plasma (*C*_*b*_), intracellular (*C*_*c*_), intracellular phosphorylated (*C*_*cp*_), and intracellular diphosphorylated (*C*_*cpp*_) tenofovir using the model of Dixit and Perelson [42] that includes drug partitioning across the cell membrane. **(b)** Example of viral load and CD4+ T cell response to TDF monotherapy with perfect adherence and cessation after 300 days. TDF-DP concentration, re-scaled from (**a**) in units of *μg/mL* along the righthand axis, is overlaid for reference. **(c)** Basic reproductive ratio of WT and drug-resistant virus at steady-state peak and trough TDF-DP concentrations, as a function CTL efficacy *p* varied with *f* to maintain a setpoint viral load of 51,000 copies/mL. **(d)** Influence of *p*, varied as in (**c**), on the time from HIV infection on TDF monotherapy until emergence of the *K65R* mutant at a viral load above 50 copies/mL. The time to resistance is 29 days at *p* = 0, and rises to ever-increasing durations as *p* reduces *R*_0_. **(d)** Influence of *p* on the time until reversion of resistance after monotherapy cessation. Reversion times range from 37 days at *p* = 0 to 15 days as *R*_0_ approaches 1.

Using a pharmacokinetic model by Dixit and Perelson [42] to translate 300 mg daily dosing of TDF into intracellular diphosphate concentrations, our model confirmed that TDF monotherapy transiently suppresses viremia, but then selects for the *K65R* mutant. Figure 3a shows our reproduction of the Dixit and Perelson pharmacokinetic model, emphasizing the importance of modeling intracellular drug species, which exhibit a longer half-life and therefore higher concentrations than those found in plasma. Figure 3b shows an example of prolonged adherence to TDF monotherapy initiated during HIV infection, with *p* set to 1.5 × 10^-5^. Consistent with experimental data, resistance begins to emerge after 50 days, with the *K65R* mutant dominating the viral population at a lower viral load and a higher CD4 count than WT infection due to the mutant’s reduced replicative capacity. This reduced fitness drives reversion to a WT-dominated infection after cessation of TDF monotherapy.

To investigate the effect of immune pressure on resistance and reversion rates, we next analyzed the effect of varying *p*, balanced with *f* (per Equation 9 and similarly to Figure 2c), on resistance and reversion times for a fixed interval of TDF monotherapy. We defined resistance as the first time *K65R* viral load reaches 50 copies/mL, and reversion as the crossover point between *K65R* and WT viremia. We analyzed a range of *p* that permits growth of the *K65R* mutant according to its *R*_*0*_ at peak and trough TDF-DP concentrations, shown in Figure 3c. Note that the pharmacokinetic model with intracellular TDF-DP ensures that the peak-to-trough change in active drug concentration only brings *R*_*0*_ of the WT above 1 for a very narrow range of *p*, illustrating the pharmacological “forgiveness” of daily TDF for most levels of immune pressure.

Increasing *p* lengthened the delay time until resistance from 29 days to ever-increasing values as *R*_*0*_ of *K65R* declined toward 1, as shown in Figure 3d. The time from monotherapy cessation until reversion to a WT-dominated infection declined with increasing *p* over the same range. Reversion required a maximum of 37 days in the absence of CTL activity with *p* set to zero. As increasing *p* drove the *R*_*0*_ of *K65R* toward 1, this duration dropped toward a minimum of 15 days (Figure 3e). Thus, increasing immune pressure led to slower appearance and more rapid reversion of the drug-resistant mutant. Intuitively, this result can be construed as a balance between immune pressure, which favors the WT due to the mutant’s fitness cost, and pharmacological pressure, which favors the mutant. In the drug-free condition shown in Figure 2b, a sufficiently high value of *p* drives the mutant *R*_*0*_ below 1, only allowing the WT to survive in the absence of drug. With daily TDF dosing, it is the mutant that can survive for an intermediate range of *p*, and this range occurs at overall lower values of *p* compared to the drug-free state as a result of pharmacological pressure exerted on both quasispecies.

The dependence of resistance/reversion rates on immune strength may have further implications on the effect of late entry into therapy, or, in the case of PrEP, whether the accidental misuse of PrEP occurred during initial or early infection (due to breakthrough infection or false negative HIV tests during the “window period” of early infection) or well into infection (due to unauthorized drug use or failure of HIV testing).

### Long-term effect of monotherapy: latent reservoirs

The net effect of increasing *p* in the short term is a shorter interval of resistance-dominated infection, i.e., fewer viral replication cycles involving the drug-resistant mutant. However, the model as used thus far allows the system to rapidly return to equilibrium after cessation of monotherapy, yielding no long-term implications of transient drug resistance. To investigate these long-term implications, we included another key feature of HIV infection: the incorporation of virus into long-lived latently infected cells capable of re-activating after months or years of suppressed viral replication.

For each mutant, we included a latently infected cell compartment *w*_*i*_, as shown in Equations 6–7 and illustrated in Figure 1c. We hypothesized that slow re-activation rates would endow this compartment with a “memory” of the virus subtypes that have undergone prior replication cycles, such that more intense and prolonged infection with drug-resistant virus would increase the fraction of drug-resistant mutants available for future re-activation. During non-suppressed viremia, the contribution of this compartment would be negligible compared to rates of forward- and back-mutation. However, during fully suppressive therapy, the latent compartment would become the sole source of new virions, and therefore would govern the production rate of drug-resistant mutant virions.

**Figure 4:**
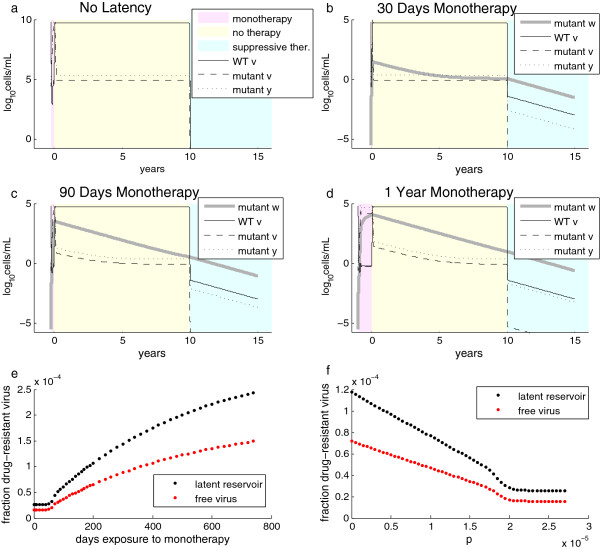
**Long-lived latently infected cells “remember” drug resistance.** Experiments begin with a variable duration of daily TDF monotherapy applied with perfect adherence (pink regions), followed by a decade without treatment (yellow region), and finally, five years of suppressive therapy (1000-fold reduction in viral replication, blue region). **(a)** In the model without latency, counts of free virus (*v*, solid line for WT and dashed line for mutant) and cells with incorporated viral DNA (*y*, dotted line for mutant) are shown for a 15-year period beginning with 90 days of daily TDF monotherapy. The system returns to equilibrium shortly after monotherapy cessation. When a latently infected compartment *w* is added, the number of cells with latently incorporated mutant virus grows throughout a monotherapy exposure of 30 days **(b)**, 90 days **(c)**, or 365 days **(d)**. For the decade following monotherapy exposure, the reservoir declines while being replaced with WT latently infected cells. When suppressive therapy is applied for the final five years, the latent reservoir provides the dominant source of virus. In (**b**), the level of mutant virus plateaus approximately 7 years after monotherapy cessation because the latent reservoir becomes a smaller source of mutants than back-mutation of replicating virus. **(e)** Longer monotherapy exposure produces more mutant virus in the latent reservoir after two years of suppressive therapy. The fraction of mutant free virions, though also increased by long monotherapy, is lower than the fraction in the latent compartment due to the reduced replicative capacity of the mutant. **(f)** For a 90-day interval of monotherapy, increasing immune pressure reduces the fraction of drug-resistant free virus and latently infected cells, but loses any incremental effect at *p*>10^-5^. This is because, similarly to (**b**), back-mutation of replicating WT virus becomes a larger source of latently incorporated mutants during the untreated (yellow) interval.

To test this hypothesis about the dynamics of the latency-embellished model, we simulated an interval of monotherapy, followed by one decade of untreated infection and, finally, an interval of prolonged suppressive therapy. The delay of one decade allows the very long-term effects of past drug resistance to be evaluated. Suppressive therapy is assumed to be a regimen against which the single mutations that exist in all infected individuals do not confer resistance, such as triple-drug highly active antirectroviral therapy (HAART). (Any given single-position mutant is almost guaranteed to exist in an infected individual due to the high error rate of reverse transcriptase, but the pre-existance of a mutant resistant to all components of HAART is unlikely [32]). Rather than modeling the pharmacokinetics of all three drugs in detail, we used a simple caricature for suppressive therapy in which we reduced the burst size of the virus by 1000-fold. As shown in Figure 4a, this rapidly drove the free virus and the activated, productively infected cell populations toward extinction. When the long-lived latent compartment *w* was added, as in Figure 4b-d, the latent reservoir maintained a very low-level viremia, representing the source of potential re-activation of virus that is observed after treatment cessation or failure.

The level of drug-resistant mutants present in this compartment (*w*_*mutant*_) grows during monotherapy when replication of the mutant is favored over that of the wild-type, and declines in the absence of monotherapy while being gradually replaced with wild-type latently infected cells (*w*_*WT*_) until it reaches its equilibrium concentration. At equilibrium, both the latent and the active infected cell populations are primarily maintained through forward mutation of the wild-type virus due to its error rate *q*. When only 30 days of monotherapy are used to initially grow *w*_*mutant*_, this equilibrium level is reached within a decade, as shown in Figure 4b. When monotherapy is applied for longer intervals, such as 90 days (Figure 4c) or 1 year (Figure 4d), *w*_*mutant*_ requires over a decade to reach equilibrium. Thus, as expected and shown in Figure 4e (graphed at the values achieved after 2 years of suppressive therapy), the fraction of the latent reservoir harboring drug resistance during the final period of suppressive therapy increases with increasing duration of initial monotherapy, except for short monotherapy exposures (<50 days) for which equilibrium is reached during the intervening decade. This threshold time would be shorter for shorter intervening spans between monotherapy and suppressive therapy. The fraction of drug-resistant free virus produced from the latent reservoir exhibits the same trend as the fraction contained in the latent reservoir, but is lower due to the reduced replicative capacity of the mutant, as shown in Figure 4f.

The previously discussed examples were conducted with immune pressure *p*, set to 1.5 × 10^-5^. As a final step in analyzing the behavior of our extended model with latency, we varied *p*, which reduced the fraction of drug-resistant mutant in the latent compartment (Figure 4g) and in the free virus produced by the latent compartment (Figure 4h). As with the monotherapy duration, the trends were the same for latently infected cells and free virus produced, but the fraction of mutant virus was lower in the free virus pool due to the reduced replicative capacity of the mutant. At a sufficiently large *p*, no further reduction in the fraction of mutant was observed. Similar to the minimum duration of monotherapy required for variation in Figure 4e-f, this threshold occurs because back-mutation of the wild-type virus became the primary source of drug-resistant mutants.

We hypothesize that incorporation of drug-resistant virus into the long-lived latent reservoir could explain why past exposure to subclinical drug concentrations (e.g., single-dose nevirapine administration for prevention of mother-to-child-transmission) can lead to increased risk of treatment failure long after the initial drug exposure has waned and any rapidly-replicating viral populations should have returned to equilibrium. Thus, minimizing the total exposure to drug-resistant viremia would reduce the incorporation of these mutants into the latent pool.

### Patterns of adherence: effect on drug resistance dynamics

We have already learned that increased immune pressure can reduce exposure to resistance by delaying its onset and hastening its reversion. However, the pattern of adherence to a drug regimen, including accidental PrEP during infection, is another important source of heterogeneities that may influence the total exposure the drug resistance and thus influence the long-term outcome. We therefore finish with an exploration of drug adherence patterns and their effect on total exposure to drug-resistant viremia.

**Figure 5:**
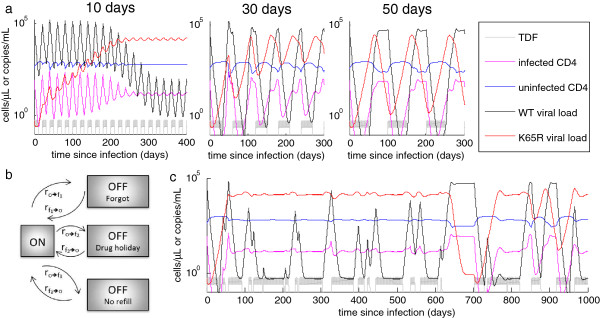
**Adherence patterns influence the dynamics of drug resistance. (a)** Dynamics of drug resistance assuming 50% adherence with periodic adherent and non-adherent spans lasting 10 days (left panel), 30 days (middle panel), or 50 days (right panel). TDF-DP concentration is overlaid in gray for reference. **(b)** Example of how a Markov model for drug adherence could represent underlying drivers of missed doses. **(c)** Dynamics of drug resistance using a two-state Markov model with exponentially distributed duration spent in the adherent and non-adherent states, each with an average duration of 30 days. This is the stochastic version of the periodic model in the middle panel of (**a**), providing an example of how such a model could be driven stochastically to reflect this aspect of real-life adherence patterns.

Unlike suppressive therapy, for which pauses in treatment lead to subclinical drug concentrations that drive resistance, accidental PrEP use during infection can drive resistance even with perfect adherence. Even so, we found that the dynamics of adherence determine the proportion of time spent with drug resistance. For a fixed fraction of doses taken, this depends on the duration of adherent and non-adherent spans another variable is the degree of periodicity or stochasticity in these durations. In the examples shown, we used a simple assumption of 50% adherence with immune pressure *p* fixed at *10*^*-5*^, a value at which resistance occurs in 37 days of constant monotherapy, and reversion after prolonged monotherapy occurs after 24 days. We compared a perfectly periodic schedule to the opposite extreme of a Markov process. For periodic 10-day transitions between adherence states, resistance cannot develop in a single span of monotherapy, but leads to eventual and sustained resistance with no opportunity for reversion (Figure 5a, left panel). Longer durations of adherence states permit reversion of resistance, leading to a smaller proportion of time spent in the resistant state (Figure 4a). In a stochastically driven model, elaborate Markov chains can be constructed with this model to represent different causes of adherence that may lead to different durations of dose-taking and dose-missing, as shown in Figure 5b. A simple, two-state Markov model with transition rates of (30 days)^-1^ allows for wide stochastic variation in the time spent in adherence states, including occasional longer intervals spent in states that favor drug resistance (Figure 5c). These longer dwell times may be the main drivers of resistance in individuals whose time to resistance is long compared to the average transition rate between states (e.g., due to a higher immune pressure *p*).

The patterns of adherence – particularly, whether reversion can occur within the intervals of non-adherence – can therefore influence the overall exposure to drug resistance even for the same total fraction of doses missed. Knowing that immune pressure influences the required time for development and reversion of resistance, the critical dwell times in the dose-taking and dose-missing states would vary among individuals, such that populations with different immune phenotypes may exhibit different responses to particular patterns of drug adherence. Through our latency model, we further hypothesize that these changes in short-term resistance dynamics may influence treatment outcomes long after the exposure to drug resistance.

## Discussion

Modern population models of HIV transmission are limited in their ability to predict the impact of drug resistance on individuals and the epidemic, because their representation of the development of drug resistance, if any, is simplistic. This simplification is chosen in part for lack of a way to model within-host dynamics in a manner that is consistent with observables such as viral load, which is heterogeneous across individuals, populations, and time periods [70]. We have shown how observable quantities such as setpoint and acute peak viral load can be independently tuned in such a model over the range of values observed in patients [53,63]. Modulation of the parameters *p* and *f* in our model can recapitulate common patterns of early viremia: rapid decline to setpoint, a slow “shoulder” with gradual decline to setpoint, or an initial interval of viral suppression followed by a rebound of viremia, which approaches setpoint after 90 days or more [53].

The kinetics of tenofovir resistance due to *K65R* mutation are not fully characterized in humans [67-69]. By accounting for adherence patterns, pharmacokinetics, and the competition between quasispecies at time-varying drug concentrations, we were able to model the dependence of resistance and reversion rates on heterogeneities in host immunity and patterns of drug adherence. At constant overall levels of adherence, we showed the relationship between the duration of dose-taking and dose-skipping intervals and the resulting proportion of time that *K65R* dominated the infection. We also examined the extremes of periodic versus exponentially distributed transitions between drug-taking and drug-missing states. Reality is likely somewhere in-between: for example, lack of a prescription refill may cause monthly transitions into a drug-missing state, yet the transition back to a drug-taking state may be more variable, depending on the accessibility of the pharmacy, availability of drugs, transportation resources, and other factors.

Without a long-lived latent compartment, our model predicted rapid reversion of resistance with no longer-term impact on the host. With fully suppressive therapy, the populations of both WT and mutant virus drop toward extinction, as back-mutation of one quasispecies can no longer contribute to sustaining the others. In this state, re-activation of long-since-incorporated latent virus becomes a significant contributor to the viral pool. By including a latent compartment as has been described [32,33], we were able to observe increasing proportions of *K65R* mutant in both the latent reservoir and the free virus population as the duration of TDF exposure increased, and as immune pressure *p* decreased.

This model feature provides a potential link between past monotherapy exposure (e.g., misuse of PrEP or single-dose nevirapine administration for prevention of mother-to-child-transmission) and future susceptibility to treatment failure. It explains why past exposure to monotherapy may increase the risk of treatment failure many years later. Further, it allows for host heterogeneities in immune response to be considered in evaluating the risk of this outcome.

Our analysis has thus far been limited to single-position mutations. In part, this is driven by the availability of detailed *in vitro* dose–response data for single-position mutants [46]. Additionally, the mutation rate of HIV is high enough that any given single-point mutant is likely to exist within a patient by the end of the acute phase. In contrast, fewer than one-third of double mutants are present during a WT-dominated infection, and a given triple-mutant is unlikely to already exist somewhere in the body [71]. In such situations, a stochastic simulation with discrete viral counts would be required to account for small populations such as rare mutants.

In the present analysis, we deliberately chose scenarios in which our continuous deterministic model would agree with the outcome of a stochastic model. We confirmed this through stochastic simulation of a subset of the experiment points. Although we found that simulations of a single milliliter did exhibit species fade-out, simulations of approximately 1% of the total system volume fully approached deterministic model results. Application of stochastic modeling to new areas in which the results could deviate from deterministic models, such as multi-position mutants, is an area of ongoing work.

## Conclusions

To date, no model has linked the population-level dynamics of HIV transmission with a representation of within-host interactions of HIV virus dynamics, host immune response, and drug PK/PD [7]. We have shown that host immunity and patterns of drug adherence are important drivers of individual-level risks and dynamics of drug adherence. Understanding how within-host models can capture among-host heterogeneities is an important step toward the milestone of bridging within-host and population-level models of HIV drug resistance.

## Competing interests

The authors declare that they have no competing interests.

## Authors’ contributions

AB and PE constructed the model, ran the model, and analyzed results. AB drafted the manuscript. AB and PE edited and approved the final manuscript.

## Supplementary Material

Additional file 1: Table S1 Sensitivity analysis of the virus dynamics model (without antiviral drugs). Parameters of the model (left column) are systematically decreased by 10%, then increased by 10%. The percent change in the steady-state values for WT viral load, WT-infected cell count, *K65R* mutant viral load, and *K65R* mutant-infected cell count are shown. Additionally, the percent change in the peak viral load value during the acute phase, and of the time of this peak, are shown. Steady-state values are the most sensitive to changes in k, λ, and u, while acute phase dynamics are most sensitive to changes in β , k, and u. Parameter values are set as in Figure 1/Table 1, with p = 1.5x10-^5^. **Table S2.** Sensitivity of resistance and reversion times to model parameters. The time of emergence of drug resistance is sensitive to the infection rate, viral production rate, and viral clearance rate (β , k, and u), as well as f, which combines changes in β and k such that steady-state viral load remains constant. In is also sensitive to the production and clearance of target cells (λ and d), clearance of infected cells (a), and the production, clearance, and killing power of immune cells (b, c, and p). Parameter values are set as in Figure 2/Table 1, with p = 1.5x10-^5^. Click here for file
