# Supplementary material for: A model of HIV drug resistance driven by heterogeneities in host immunity and adherence patterns
Source: BMC Syst Biol. 2013 Feb 4;7:11. doi: 10.1186/1752-0509-7-11 (PMC3643872; doi:10.1186/1752-0509-7-11)
Supplement: Additional file 1: Table S1 — Sensitivity analysis of the virus dynamics model (without antiviral drugs). Parameters of the model (left column) are systematically decreased by 10%, then increased by 10%. The percent change in the steady-state values for WT viral load, WT-infected cell count, K65R mutant viral load, and K65R mutant-infected cell count are shown. Additionally, the percent change in the peak viral load value during the acute phase, and of the time of this peak, are shown. Steady-state values are the most sensitive to changes in k, λ, and u, while acute phase dynamics are most sensitive to changes in β , k, and u. Parameter values are set as in Figure 1/Table 1, with p = 1.5x10^-5^. Table S2. Sensitivity of resistance and reversion times to model parameters. The time of emergence of drug resistance is sensitive to the infection rate, viral production rate, and viral clearance rate (β , k, and u), as well as f, which combines changes in β and k such that steady-state viral load remains constant. In is also sensitive to the production and clearance of target cells (λ and d), clearance of infected cells (a), and the production, clearance, and killing power of immune cells (b, c, and p). Parameter values are set as in Figure 2/Table 1, with p = 1.5x10^-5^. [file 1752-0509-7-11-S1.pdf]

Supplementary Material for:

A Model of HIV Drug Resistance Driven by Heterogeneities in Host Immunity and Adherence Patterns

Anna Bershteyn[1] and Philip A. Eckhoff[1]

[1]Epidemiological Modeling Group, Intellectual Ventures Laboratory

Supplementary Table 1. Sensitivity analysis of the virus dynamics model (without antiviral drugs). Parameters of the model (left column) are systematically decreased by 10%, then increased by 10%. The percent change in the steady-state values for WT viral load (), WT-infected cell count, K65R mutant viral load (), and K65R mutant-infected cell count are shown. Additionally, the percent change in the peak viral load value during the acute phase, and of the time of this peak, are shown. Steady-state values are the most sensitive to changes in k, λ, and u, while acute phase dynamics are most sensitive to changes in β, k, and u. Parameter values are set as in Figure 1/Table 1, with p = 1.5×10^{-5}.

	Fold change in value after this parameter increases/decreases by 10%											
	$V_{ss,WT}$		$CD4_{ss,WT}$		$V_{ss,mutant}$		$CD4_{ss,mutant}$		Acute peak *v*		Peak time	
	–	+	–	+	–	+	–	+	–	+	–	+
**β**	-4%	3%	-4%	3%	-4%	3%	-4%	3%	-5%	3%	9%	-7%
**k**	-14%	14%	-4%	3%	-14%	14%	-4%	3%	-14%	14%	8%	-7%
**p**	8%	-7%	8%	-7%	8%	-7%	8%	-7%	1%	-1%	-0.2%	0.1%
**b**	-8%	7%	-8%	7%	-8%	7%	-8%	7%	-0.2%	0.2%	-0.003%	-0.003%
**c_1**	8%	-7%	8%	-7%	8%	-7%	8%	-7%	1%	-1%	-0.2%	0.1%
**λ**	-14%	14%	-14%	14%	-14%	14%	-14%	14%	-10%	10%	3%	-3%
**d**	4%	-4%	4%	-4%	4%	-4%	4%	-4%	9%	-9%	-3%	3%
**u**	15%	-12%	4%	-4%	15%	-12%	4%	-4%	13%	-12%	-5%	6%
**a**	7%	-6%	7%	-6%	7%	-6%	7%	-6%	5%	-5%	-1%	1%

Supplementary Table 2. Sensitivity of resistance and reversion times to model parameters. The time of emergence of drug resistance is sensitive to the infection rate, viral production rate, and viral clearance rate (β, k, and u), as well as f, which combines changes in β and k such that steady-state viral load remains constant. In is also sensitive to the production and clearance of target cells (λ and d), clearance of infected cells (a), and the production, clearance, and killing power of immune cells (b, c_1, and p). Parameter values are set as in Figure 2/Table 1, with p = $1.5x10^{-5}$.

Parameter	Fold change in **resistance** time when param		Fold change in **reversion** time when param	
	Decreases by 10%	Increases by 10%	Decreases by 10%	Increases by 10%
**β**	51%	-24%	-7%	5%
**k**	122%	-34%	-14%	8%
**p**	-20%	33%	8%	-8%
**b**	38%	-19%	-9%	8%
**c_1**	-20%	33%	8%	-8%
**λ**	47%	-22%	-9%	6%
**d**	-19%	34%	5%	-6%
**u**	-26%	44%	6%	-7%
**a**	-19%	29%	7%	-7%
**g**	-0.1%	0.1%	0.000%	0.000%
**h**	0.1%	-0.1%	0.8%	-0.5%
**f**	-18%	26%	4%	-4%
**K_a**	-1%	0.8%	-0.1%	0.1%
**K_e**	2%	-2%	0.3%	-0.3%
**K_{1f}**	-4%	3%	-0.4%	0.4%
**K_{1b}**	3%	-2%	0.4%	-0.3%
**K_{2f}**	-3%	3%	-0.4%	0.4%
**K_{2b}**	3%	-3%	0.4%	-0.3%
**K_m**	0.003%	0.002%	0.1%	0.1%
**K_c**	2%	-2%	3%	-2%
**H**	-4%	4%	-0.5%	0.4%
**$f_{plasma\text{-}bound}$**	0.2%	-0.3%	0.1%	-0.02%
**$IC_{50,WT}$**	0.4%	-0.7%	4%	-4%
**$IC_{50,mut}$**	9%	-6%	-2%	1%
**m_{WT}**	-2%	0.6%	-6%	4%
**m_{mut}**	-7%	8%	1%	-2%
